# Mapping kommunaler psychosozialer Angebote für Kinder und Eltern: Zugangs- und Steuerungsbarrieren in der präventiven Versorgung – eine Mixed-Methods-Studie

**DOI:** 10.1007/s00103-026-04307-6

**Published:** 2026-09-11

**Authors:** Sarah Beierle, Anika Reinhart, Susanne Kuger, Sabine Walper, Birgit Reißig, Silvia Schneider, Lukka Popp, Babett Voigt, Freia De Bock

**Affiliations:** 1 Deutsches Jugendinstitut (DJI) München und Außenstelle Halle (Saale), Haus 12/13, Franckeplatz 1, 06110 Halle (Saale), Deutschland; 2 Fakultät für Psychologie und Pädagogik, Ludwig-Maximilians-Universität München, München, Deutschland; 3 Mitglied der Freien Universität Berlin und Humboldt-Universität zu Berlin, Institut für Medizinische Soziologie und Rehabilitationswissenschaft, Charité – Universitätsmedizin Berlin, Berlin, Deutschland; 4 Forschungs- und Behandlungszentrum für psychische Gesundheit (FBZ), Ruhr-Universität Bochum, Bochum, Deutschland; 5 Standort Bochum-Marburg, Deutsches Zentrum für psychische Gesundheit (DZPG), Bochum-Marburg, Deutschland

**Keywords:** Psychosoziale Angebotslandschaft, Sozial benachteiligte Stadtteile, Angebotsmapping, Kommunale Koordination, Transparenz von Angeboten, Psychosocial service landscape, Socially disadvantaged city districts, Service mapping, Municipal coordination, Service transparency

## Abstract

**Hintergrund:**

Angebote der Primär- und Sekundärprävention psychischer Erkrankungen bei Kindern verteilen sich auf die Sektoren Gesundheit, Bildung und Soziales. Umfassende Prävention erfordert deren koordiniertes Zusammenwirken auf kommunaler Ebene. Bislang fehlt eine systematische wissenschaftliche Erfassung der kommunalen psychosozialen Angebotslandschaft für Familien.

**Methoden:**

In 2 sozial benachteiligten Stadtteilen einer west- und einer ostdeutschen Großstadt wurden im Zeitraum 02–07/2024 Fachkräfte aus den Sektoren Gesundheit, Bildung und Soziales interviewt (*N* = 19) und psychosoziale Präventionsangebote anschließend intersektoral online gemappt. Die Angebote wurden nach Unterstützungsfunktionen kategorisiert und mit den Interviewergebnissen rückgekoppelt.

**Ergebnisse:**

Die Fachkräfte beschrieben die Angebotslandschaft als fragmentiert und unübersichtlich; Informations- und Navigationsbarrieren betrafen besonders einkommensarme und zugewanderte Familien. Beim Mapping wurden 122 Einrichtungen mit 360 online sichtbaren Angeboten identifiziert. Es zeigte sektorale Schwerpunkte, Hinweise auf potenzielle Unterversorgung in der spezialisierten Kindermedizin, Kinder- und Jugendlichenpsychotherapie sowie Kinder- und Jugendpsychiatrie, eine geringe Ansprache von Migrant:innen und Unterschiede in der digitalen Auffindbarkeit.

**Diskussion:**

Das Mixed-Methods-Vorgehen bildet eine Grundlage für die Entwicklung eines Instruments zur systematischen Erfassung psychosozialer Angebotslandschaften. Beim Mapping wurden jedoch nur online sichtbare Angebote erfasst und bislang weder Wartezeiten, weitere Zugangshürden noch die tatsächliche Nutzung abgebildet. Künftig sollten diese Aspekte berücksichtigt und Fachkräfte kontinuierlich in die Instrumentenentwicklung einbezogen werden.

**Zusatzmaterial online:**

Zusätzliche Informationen sind in der Online-Version dieses Artikels (https://doi.org/10.1007/s00103-026-04307-6) enthalten.

## Abstract (en)

**Background:**

Services for the primary and secondary prevention of mental disorders in children are distributed across the health, education and social sectors. Comprehensive prevention requires their coordinated collaboration at the municipal level. However, there is currently no systematic scientific assessment of municipal psychosocial service landscapes for families.

**Methods:**

In two socially disadvantaged neighbourhoods in a large city in western Germany and one in eastern Germany, professionals from the health, education and social sectors were interviewed (*N* = 19), and psychosocial prevention services were mapped online across sectors. Services were categorised according to support functions, and the results were fed back into the interview findings.

**Results:**

Professionals described the service landscape as fragmented and difficult to navigate; information and navigation barriers particularly affect low-income and immigrant families. The mapping identified 122 facilities providing 360 services visible online. It revealed sector-specific patterns; indications of potential undersupply in specialised paediatrics, child and adolescent psychotherapy, and child and adolescent psychiatry; limited outreach to migrants; and differences in the digital findability of services.

**Discussion:**

The mixed-methods approach provides a basis for developing an instrument for the systematic assessment of psychosocial service landscapes. However, the mapping captured only services visible online and has not yet included waiting times, further barriers to access or actual service use. Future instruments should address these aspects in greater detail and involve professionals in the development of the instrument.

## Hintergrund

Die psychosoziale Versorgung von Kindern und Familien ist Bestandteil der staatlichen Daseinsvorsorge und umfasst insbesondere Angebote der Primär- und Sekundärprävention, die psychischen Belastungen vorbeugen bzw. deren frühzeitige Bewältigung fördern sollen, bevor manifeste Erkrankungen entstehen [1, 2]. Solche Angebote finden im medizinischen System, in der Kinder- und Jugendhilfe, im Bildungswesen sowie in weiteren sozialen Unterstützungsstrukturen statt und bilden gemeinsam eine psychosoziale Präventions- und Angebotslandschaft mit dem Potenzial, soziale Teilhabe und Chancengerechtigkeit zu verbessern [3].

Diese Angebotslandschaft ist besonders dort relevant, wo Familien vielfältigen psychosozialen Stressoren wie Armut, Arbeitslosigkeit oder elterlichen psychischen Erkrankungen ausgesetzt sind. Gerade für belastete Familien ist entscheidend, dass Angebote niedrigschwellig zugänglich und sektorenübergreifend vernetzt sind, damit Zugänge nicht vom individuellen Informations- und Suchverhalten abhängen [4].

Fachkräfte im Gesundheits‑, Bildungs- und Sozialsektor stehen vor der Aufgabe, psychische Auffälligkeiten früh zu erkennen, Verweiswege zu kennen und bestehende Angebote hinsichtlich ihrer Passung und Wirksamkeit einschätzen zu können [5]. Ein koordiniertes, systemübergreifendes Zusammenwirken kann hierbei effiziente präventive Pfade eröffnen, wie sie etwa im Konzept der „Präventionsketten“ angelegt sind [6].

Psychosoziale Prävention erfordert somit eine sektorenübergreifende Perspektive und einen systematischen Überblick über lokal verfügbare Unterstützungsstrukturen, um Fachkräfte und Familien zu befähigen, die regionale Angebotslandschaft gezielt zu nutzen und Zugänge zu Hilfen zu erleichtern. Im Unterschied zu klassischen Ansätzen der Patientensteuerung, die vorrangig behandlungsbedürftige Fälle und etablierte Versorgungspfade im Gesundheitssystem fokussieren [7, 8], richtet sich der hier verfolgte Ansatz auf eine vorgelagerte Ebene präventiver Hilfen.

Unter präventiver Klientensteuerung verstehen wir die systematische Kapazität des Systems von Angeboten und Fachkräften, Familien bei psychosozialem Unterstützungsbedarf frühzeitig zu identifizieren und sektorenübergreifend zu passenden Angeboten zu lotsen.

Unserer Kenntnis nach ist die psychosoziale Angebotslandschaft in deutschen Kommunen bislang noch nicht sektorenübergreifend wissenschaftlich systematisch erfasst worden. Vorhandene internationale Angebotsmappings fokussieren auf spezifische Krankheitsbilder, Zielgruppen oder isolierte Versorgungsbereiche [1, 9, 10] und erlauben daher nur eingeschränkte Darstellungen der lokalen Angebotslandschaft. Ein umfassendes Angebotsmapping, das auf zuvor erhobenen Perspektiven von Fachkräften aufbaut, kann ein Ausgangspunkt sein, um Angebotslandschaften über Sektoren hinweg besser zu verstehen und kommunale Steuerungs- und Planungsprozesse datenbasiert zu unterstützen.

Hier wird ein erster explorativer Versuch vorgestellt, mittels einer Mixed-Methods-Studie die Angebotslandschaft auf kommunaler Ebene darzustellen. Die Befunde zielen nicht auf Generalisierung ab, sondern auf die Entwicklung eines Ansatzes, der für weitere Forschung und kommunale Planung herangezogen werden kann. Die übergeordnete Forschungsfrage lautet: Wie können psychosoziale Präventionsangebote für Kinder im Grundschulalter und deren Familien auf kommunaler Ebene systematisch erfasst werden? Zur Beantwortung werden folgende Teilfragestellungen untersucht:

- Welche Barrieren sehen Fachkräfte in der psychosozialen Versorgung vor Ort?
- Wie lässt sich die psychosoziale Angebotslandschaft für Kinder im Kita- und Grundschulalter und ihre Eltern in den untersuchten Stadtteilen beschreiben?
- Wie könnten die Angebote systematisch in Hinblick auf ihre jeweilige Funktion kategorisiert werden?
- Wie gestalten sich die Online-Sichtbarkeit und Transparenz der psychosozialen präventiven Angebote?

## Methoden

Die Studie wurde im Rahmen des Projekts „Mapping der systemübergreifenden, präventiven Unterstützungslandschaft zur psychischen Gesundheit von Kindern“ (Förderzeitraum 2023–2025) am Standort Bochum-Marburg des Deutschen Zentrums für Psychische Gesundheit (DZPG) in Kooperation mit dem Universitätsklinikum Düsseldorf, dem Deutschen Jugendinstitut und der Ruhr-Universität Bochum durchgeführt. Bei den Untersuchungsregionen handelte es sich um 2 anonymisierte sozial benachteiligte Stadtteile einer west- und einer ostdeutschen Großstadt.

Es wurde ein sequenzielles Mixed-Methods-Design mit methodischer Triangulation eingesetzt (siehe [11]). Aufbauend auf qualitativen Interviews mit Fachkräften wurden eine systematische Online-Recherche kommunaler Angebotsstrukturen und eine quantitative angebots- und vernetzungsbezogene Befragung von Fachkräften durchgeführt. Der vorliegende Beitrag fokussiert auf die qualitativen Ergebnisse zur Perspektive der Fachkräfte auf bestehende Versorgungsstrukturen und deren Übersichtlichkeit sowie auf die Ergebnisse einer systematischen Online-Recherche (Online-Desk-Research) und Kategorisierung der bestehenden Angebote (Angebotsmapping). Qualitative Ergebnisse zu Zugangsbarrieren sind bereits publiziert [12]. Ergebnisse zur sektorenübergreifenden Vernetzung werden separat veröffentlicht.

Von Februar bis Juli 2024 wurden leitfadengestützte Interviews mit Fachkräften aus den Sektoren Gesundheit, Bildung und Soziales durchgeführt (*N* = 19, beide Untersuchungsregionen). Über ein gezieltes Sampling (Purposive Sampling) wurden Fachkräfte aus unterschiedlichen Sektoren, Handlungsfeldern und institutionellen Kontexten einbezogen, die über Einblicke in Zugangswege, Versorgungsprobleme und Unterstützungsangebote für Kinder und Familien verfügten. Die Auswertung erfolgte inhaltsanalytisch nach Kuckartz [13].

Im Online-Desk-Research (Juli bis November 2025) wurden kommunale Webseiten, Trägerhomepages und Online-Verzeichnisse anhand erster Arbeitsdefinitionen (Tab. 1) systematisch ausgewertet, um Angebote und Einrichtungen aus den 3 Sektoren zu identifizieren.^1^ Da alle in den Expertenbefragungen genannten Angebote auch über die Onlinesuche gefunden werden konnten, konzentriert sich das Mapping im Folgenden primär auf die Ergebnisse des Online-Desk-Research.

**Tab. 1:** Arbeitsdefinitionen für psychosoziale Angebote, die fokussierte Zielgruppe und die untersuchten Sektoren mit dazugehörigen Angeboten

*Psychosoziale Angebote*	
= Maßnahmen und Strukturen zur Stärkung individueller und sozialer Ressourcen, zur Förderung sozialer Teilhabe und zur Unterstützung einer gesunden Lebensführung. Ziel ist es, die Bewältigungsfähigkeiten der Zielgruppen zu stärken und soziale und/oder psychische Belastungen zu reduzieren [14, 15]. Der Fokus des Mappings liegt auf primär- und sekundärpräventiven Angeboten	
Primärprävention	Maßnahmen vor Auftreten einer Erkrankung oder manifesten psychosozialen Problemlagen, mit dem Ziel, Risiken gar nicht erst entstehen zu lassen, Ressourcen zu stärken und die Inzidenz neuer Störungsfälle zu senken (z. B. Resilienzförderung, Elternkurse, soziale Kompetenztrainings; [2])
Sekundärprävention	Maßnahmen zur Früherkennung und frühzeitigen Behandlung bzw. Beratung bei Frühstadien oder klar identifizierbaren Risikokonstellationen (z. B. Screenings auf depressive Symptome, Früherkennung von Risikofamilien, Kurzinterventionen nach ersten Auffälligkeiten), um Chronifizierung und schwere Verläufe zu vermeiden [2]
*Fokussierte Zielgruppe*	
= Angebote, die sich an Kinder im Kita- und Grundschulalter und/oder an deren Eltern richten	
*Sektoren*	
Bildung	Angebote, die an Kindertageseinrichtungen und Grundschulen verortet sind
Gesundheit	Angebote klinischer Einrichtungen, kinder- und jugendärztlicher sowie psychotherapeutischer Praxen und nichtärztlicher Gesundheitsberufe
Soziales	Angebote von Trägern der Kinder- und Jugendhilfe, der freien Wohlfahrtspflege, zivilgesellschaftlichen Initiativen und anderen sozialen Diensten

Die ermittelten Angebote wurden mithilfe von im Projekt iterativ entwickelten Kategorien analysiert und funktional zugeordnet. Die Kategorien möglicher Angebotsfunktionen (s. Onlinematerial 1) wurden auf Grundlage unterschiedlicher konzeptioneller Referenzen entwickelt und in mehreren Feedbackschleifen im interdisziplinären Projektteam im Rahmen strukturierter Reflexions- und Diskussionsrunden (Sounding-Board-Meetings) konsentiert. Dabei war pädiatrische, psychologische, pädagogische und sozialwissenschaftliche Expertise vertreten. Da bislang kein entsprechendes Kategoriensystem für psychosoziale präventive Angebote vorlag, wurden die Funktionskategorien im Wechselspiel zwischen theoretischen Konzepten [2, 16] und dem empirischen Material entwickelt. Sie ermöglichen eine aggregierte Darstellung unterschiedlich benannter und komplexer Angebote und beschreiben aus einer systemischen Perspektive mögliche Funktionen innerhalb der psychosozialen Prävention.

### Beschreibung der Untersuchungsregionen

Die 2 untersuchten Stadtteile (Stadtteil 1 und Stadtteil 2) liegen in einer westdeutschen (Stadt 1) und einer ostdeutschen Großstadt (Stadt 2) mit jeweils max. 400.000 Einwohner:innen. Beide Stadtteile sind räumlich klar begrenzt und deutlich dichter bebaut und besiedelt als die jeweiligen Gesamtstädte.

Die Stadtteile sind deutlich kinderreicher als ihre jeweilige Gesamtstadt und verzeichnen im Vergleich zum jeweiligen Stadtdurchschnitt erhöhte Anteile von Kindern in SGB-II-Bedarfsgemeinschaften, was auf eine ausgeprägte soziale Belastung und einen besonderen Bedarf an niedrigschwelligen Unterstützungs- und Präventionsangeboten für Familien hindeutet. Stadtteil 1 ist deutlich kleiner, die Anzahl der Kinder in Stadtteil 2 mehr als doppelt so hoch. Stadtteil 1 weist einen hohen Anteil an Bewohner:innen mit Migrationshintergrund auf, von denen viele mittlerweile die deutsche Staatsbürgerschaft besitzen. In Stadtteil 2 hat die Zuwanderung insbesondere Mitte der 2010er-Jahre stark zugenommen. Eine Übersicht zentraler sozialstruktureller Merkmale der Untersuchungsregionen^2^ findet sich in Onlinematerial 2.

## Ergebnisse

### Die Perspektive der Fachkräfte

Im Folgenden wird auf 7 Zitate von Fachkräften verwiesen, die im Onlinematerial 3 zu finden sind.

In den Interviews beschreiben Fachkräfte der 3 Sektoren die lokale psychosoziale Angebotslandschaft als dynamisch, fragmentiert und schwer überschaubar [12]. Orientierungsprobleme bestehen vor allem bei Angeboten außerhalb des eigenen Sektors (vgl. Zitat 1). Mehrfach wird deutlich, dass sowohl Fachkräfte als auch Familien Schwierigkeiten haben, sich einen verlässlichen Überblick über Zuständigkeiten, Zugangswege und Unterstützungsangebote zu verschaffen. Zugleich bestehe der Wunsch nach mehr Bildung, Aufklärung und Weiterbildung, um unterschiedliche Zielgruppen besser erreichen und unterstützen zu können (Zitat 2).

Viele Familien seien auf aktive Hilfe von Fachkräften bei Auswahl und Koordination von Hilfen angewiesen, da sie sich nur schwer selbst informieren oder ihre Kinder zu passenden Stellen bringen könnten (Zitat 3). Aus Fachkräftesicht betrifft dies insbesondere Familien mit begrenzten zeitlichen, finanziellen oder sprachlichen Ressourcen, bei denen die Nutzung vorhandener Angebote nicht allein an deren Existenz, sondern auch an ihrer praktischen Auffindbarkeit und Erreichbarkeit scheitern kann. Dies verweist auf begrenzte Informations- und Bewältigungsressourcen im Umgang mit einem komplexen Hilfesystem.

Zudem berichten die Fachkräfte über Engpässe in der gesundheitlichen Versorgung, insbesondere bei Psychotherapie, Kinderarztpraxen und Kinder- und Jugendpsychiatrie. Lange Wartezeiten und weite Wege erschweren den Zugang, vor allem für Familien mit geringen Ressourcen (Zitat 4). Als weitere Lücken nennen die Interviewten alltagsnahe Förder- und Teilhabeangebote, die für einkommensschwache Familien häufig nur begrenzt verfügbar sind (Zitat 5).

Auch der Zugang für zugewanderte Familien bleibt herausfordernd, vor allem wegen sprachlicher Barrieren und erschwerter Zielgruppenerreichung (Zitate 6 und 7). Die Aussagen deuten darauf hin, dass migrationsbezogene Bedarfe aus Sicht der Fachkräfte nicht auf einzelne spezialisierte Angebote begrenzt sind, sondern verschiedene Unterstützungskategorien betreffen, etwa Beratung, Information und alltagsnahe Begleitung. Als relevante Voraussetzungen für die Inanspruchnahme der Angebote werden insbesondere sprachliche Verständigung, kultursensible Ansprache und niedrigschwellige Informationsvermittlung genannt.

Insgesamt wird ein strukturierter Überblick über die Angebote in den verschiedenen Sektoren vermisst, sowohl von Fachkräften für ihre eigene Arbeit als auch für hilfesuchende Familien. Die qualitativen Befunde verweisen zudem auf Versorgungsengstellen (migrations- und integrationsbezogene Unterstützungsangebote, Freizeit- und Teilhabeangebote, gesundheitsbezogene Leistungen), die im anschließenden Mapping näher betrachtet werden.

### Einrichtungen und Angebote in den Stadtteilen

#### Sektorale Verteilung der erfassten Einrichtungen.

In Stadtteil 1 wurden *n* = 51 Einrichtungen und in Stadtteil 2 *n* = 71 Einrichtungen mit psychosozialen Angeboten erfasst. Im Vergleich zeigen sich unterschiedliche sektorale Schwerpunkte bei den online auffindbaren Einrichtungen.

**Abb. 1:**
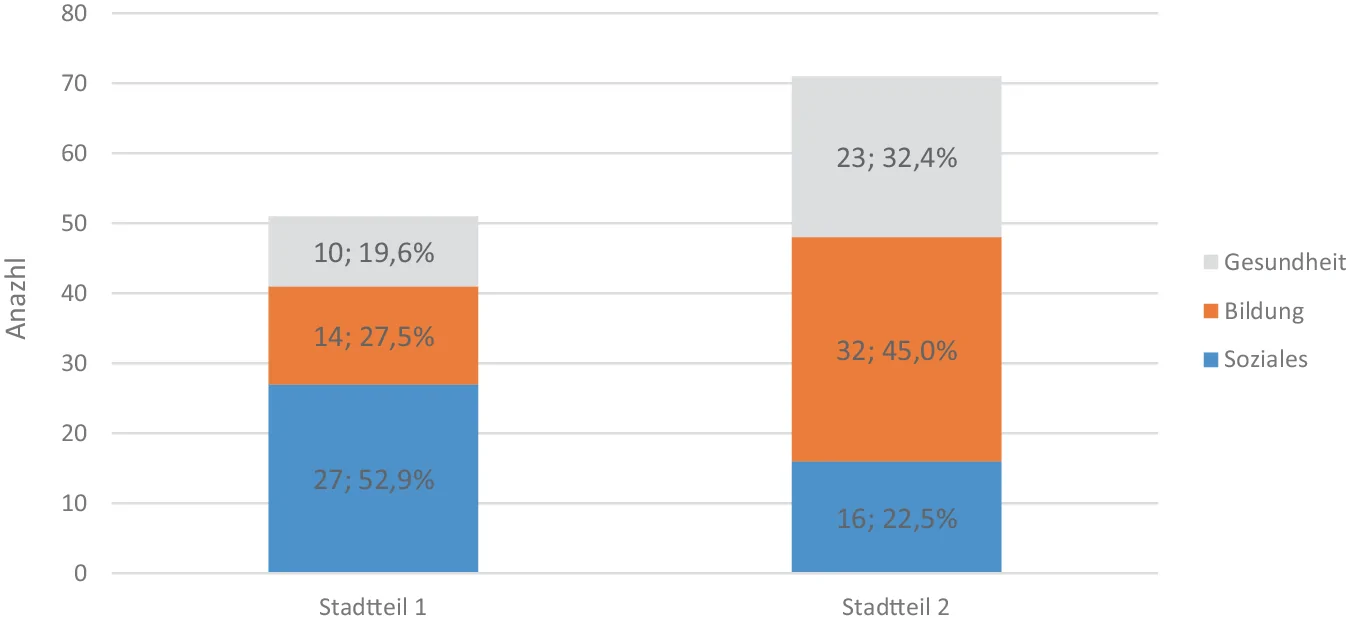
Sektorale Verteilung der online auffindbaren Einrichtungen in den untersuchten Stadtteilen: Dargestellt sind die Anzahl der Einrichtungen sowie deren Anteil an allen im jeweiligen Stadtteil erfassten Einrichtungen (Stadtteil 1: *n* = 51; Stadtteil 2: *n* = 71)

In Stadtteil 1 entfällt rund ein Fünftel der Einrichtungen (19,6 %) auf den Gesundheitssektor, gut ein Viertel (27,5 %) auf den Bildungssektor und über die Hälfte (52,9 %) auf den Sozialsektor (Abb. 1). Das lokal auffindbare Angebot konzentriert sich hier deutlich auf den Sozialsektor, der ein breites Spektrum an Beratungs‑, Unterstützungs- und Freizeitangeboten umfasst.

In Stadtteil 2 entfallen auf den Gesundheitssektor 32,4 % der Einrichtungen, auf den Bildungssektor 45,0 % der Einrichtungen und 22,5 % auf den Sozialsektor. Damit ist der Anteil von Einrichtungen aus dem Gesundheits- und insbesondere aus dem Bildungssektor deutlich höher als in Stadtteil 1, während der Sozialsektor einen wesentlich geringeren Anteil ausmacht. Im Gesundheitssektor ist Stadtteil 2 – auch unabhängig von Angeboten, die mit der höheren Kinderzahl zusammenhängen – besser ausgestattet, insbesondere im Hinblick auf die kinderärztliche Versorgung und therapeutische Gesundheitsberufe wie Ergo- und Logopädie; insgesamt wurden hier mehr als doppelt so viele Gesundheitseinrichtungen wie in Stadtteil 1 identifiziert.

Im Bildungssektor verfügt Stadtteil 2 über mehr Kitas und Grundschulen, was mit der höheren Kinderzahl zusammenhängt; hinzu kommen Hortangebote, die in Stadtteil 1 traditionell nicht vertreten sind [17]. Gleichzeitig zeigt sich in beiden Stadtteilen eine Unterversorgung im Bereich kinder- und jugendpsychiatrischer sowie stationärer pädiatrischer Angebote: Weder Kliniken der Kinder- und Jugendpsychiatrie noch Kinderkliniken oder Kinder- und Jugendpsychiater:innen sind in den Untersuchungsregionen selbst verortet und müssen außerhalb der Stadtteile aufgesucht werden.

### Sektorale Verteilung der erfassten Angebote.

In Stadtteil 1 wurden insgesamt 216 online auffindbare Angebote erfasst, in Stadtteil 2 dagegen 144. Trotz weniger Einrichtungen waren damit in Stadtteil 1 mehr Angebote online auffindbar als in Stadtteil 2.

**Abb. 2:**
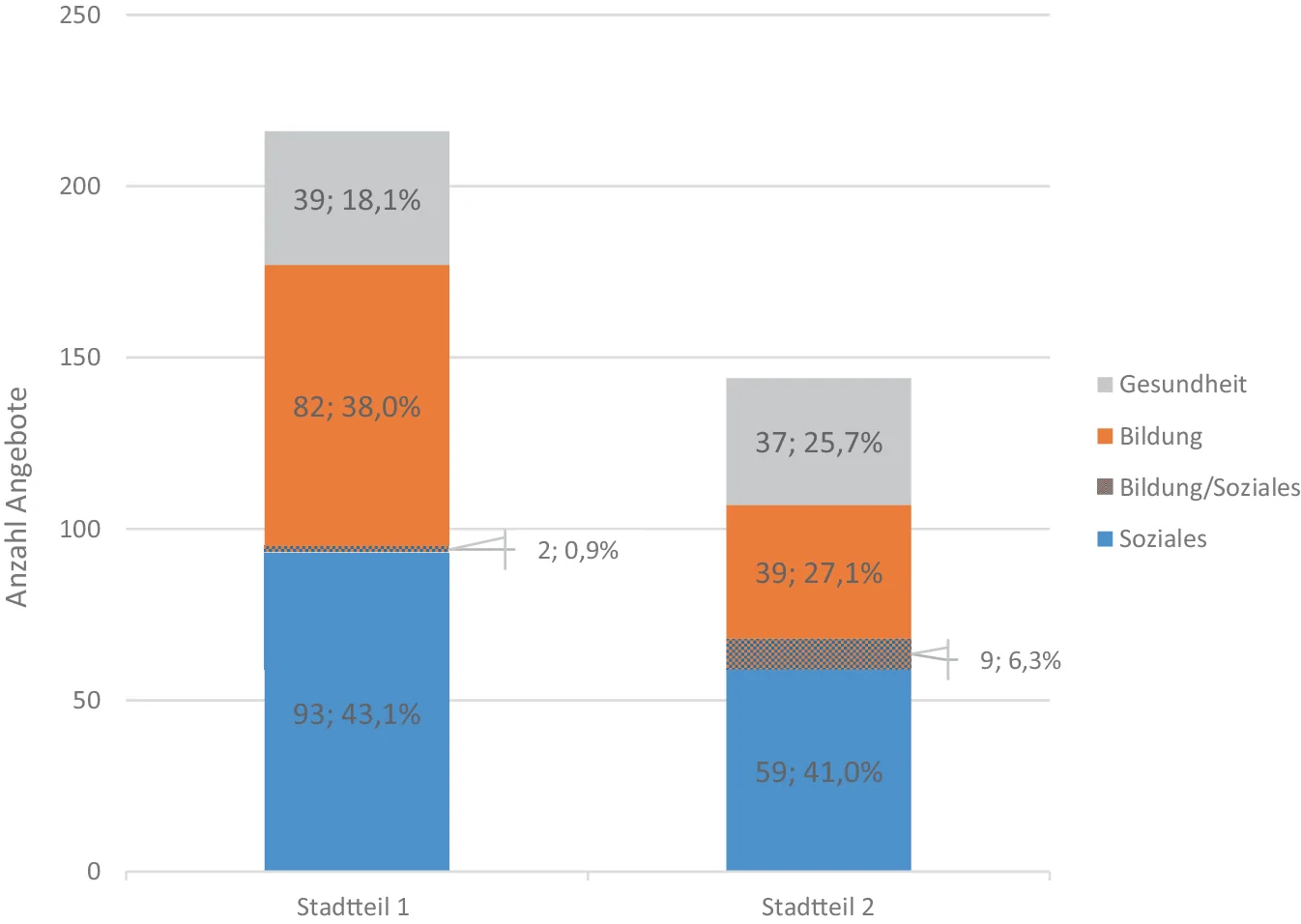
Sektorale Verteilung der online auffindbaren Angebote in den untersuchten Stadtteilen: Dargestellt sind die Anzahl der Angebote sowie deren Anteil an allen im jeweiligen Stadtteil erfassten Angeboten (Stadtteil 1: *n* = 216; Stadtteil 2: *n* = 144)

In beiden Stadtteilen ist der Sozialsektor bei den online erfassbaren Angeboten (Stadtteil 1: 43,1 %; Stadtteil 2: 41,0 %) am stärksten vertreten (Abb. 2). Kita- und Schulsozialarbeit wurden einem vierten Sektor „Bildung/Soziales“ zugeordnet, da es sich um Brückenangebote handelt; in Stadtteil 1 konnten nur 2, in Stadtteil 2 9 dieser Angebote identifiziert werden. Die Sichtbarkeit von Angeboten hängt stark von der Ausführlichkeit der Webseiten ab. Tab. 2 zeigt, dass in Stadtteil 1 durchschnittlich mehr Angebote pro Einrichtung erfasst wurden als in Stadtteil 2, insbesondere im Bildungssektor.

**Tab. 2:** Durchschnittliche Anzahl der gemappten Angebote pro Einrichtung in den 3 Sektoren

	Stadtteil 1	Stadtteil 2
Gesundheit	3,9	2,1
Bildung	6,0	1,5
Soziales	3,4	3,6

Dies legt nahe, dass die Angebote in Stadtteil 1 insgesamt ausführlicher online dokumentiert sind als in Stadtteil 2. In Stadtteil 2 verfügen im Sozialsektor einzelne Einrichtungen über keine eigene Website, im Gesundheitssektor wurden zahlreiche Praxen ohne Webauftritt identifiziert, sie waren lediglich in Ärzteverzeichnissen gelistet. Im Bildungsbereich werden zahlreiche Kitas in Sammelprofilen des Trägers mit nur begrenzt detaillierter Angebotsdarstellung geführt. Dadurch sinkt die Zahl der im Online-Desk-Research identifizierbaren Einzelangebote und es besteht die Gefahr, die tatsächliche Angebotsstruktur – insbesondere in Stadtteil 2 – zu unterschätzen.

### Funktionen der Angebote

**Abb. 3:**
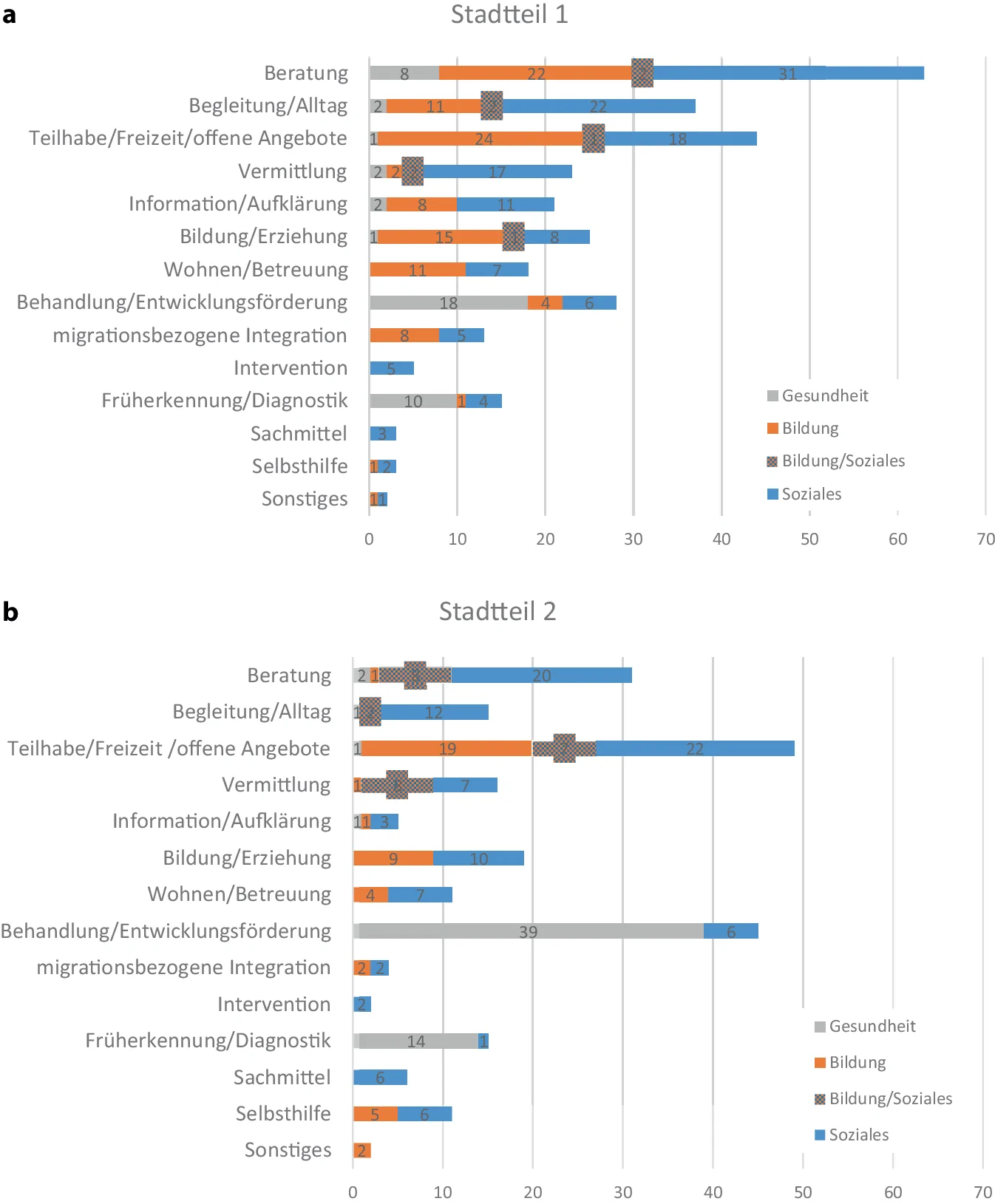
Anzahl der online auffindbaren psychosozialen Angebote nach Funktionskategorien und Sektoren in den untersuchten Stadtteilen: **a** Stadtteil 1 *n* = 303, **b** Stadtteil 2 *n* = 225. Ein Angebot konnte mehreren Funktionskategorien zugeordnet werden

Die Prozentwerte beziehen sich auf die Gesamtzahl der im jeweiligen Stadtteil auf Grundlage der online verfügbaren Angebotsinformationen vorgenommenen Funktionszuordnungen (Stadtteil 1: *n* = 303; Stadtteil 2: *n* = 225). Einem Angebot konnten bis zu 4 Funktionen zugeordnet werden. Die absoluten Häufigkeiten der Funktionszuordnungen sind in Abb. 3a, b dargestellt. Für eine Übersicht über die Funktionskategorien siehe Onlinematerial 1.

Zu den am häufigsten vertretenen Funktionen in beiden Stadtteilen zählen Beratung (Stadtteil 1: 20,8 %; Stadtteil 2: 13,8 %), Teilhabe/Freizeit/offene Angebote (Stadtteil 1: 14,9 %; Stadtteil 2: 20,9 %) sowie Begleitung/Alltag (Stadtteil 1: 12,2 %; Stadtteil 2: 6,7 %).

In Stadtteil 1 entfallen auf diese 3 Funktionen 47,9 % aller Zuordnungen. Es folgen Behandlung/Entwicklungsförderung (9,2 %), Bildung/Erziehung (8,3 %), Vermittlung (7,9 %) sowie Information/Aufklärung (6,9 %). Selbsthilfe, Sachmittelausgabe, Intervention oder Sonstiges spielen nur eine untergeordnete Rolle. Die häufigsten Funktionen liegen überwiegend im Sozial- und Bildungssektor, während der Gesundheitssektor vor allem in den Bereichen Behandlung/Entwicklungsförderung und Früherkennung/Diagnostik vertreten ist.

In Stadtteil 2 prägen Teilhabe/Freizeit/offene Angebote (20,9 %), behandlungs- und entwicklungsfördernde Leistungen (20,0 %) sowie Beratung (13,8 %) die Funktionsstruktur. Nachgeordnet folgen Bildung/Erziehung (8,4 %), Begleitung/Alltag und Früherkennung/Diagnostik (je 6,7 %) sowie Vermittlung (5,3 %). Intervention, migrationsbezogene Integrationsangebote, Sachmittel und Sonstiges treten nur vereinzelt auf. Die Funktion Teilhabe/Freizeit/offene Angebote konzentriert sich überwiegend im Bildungs- und Sozialsektor, die Funktion Behandlung/Entwicklungsförderung im Gesundheitssektor, während Beratung vor allem im Sozial- und Bildungssektor verortet ist.

## Diskussion

Durch die Entwicklung von Kategorien für die im Online-Mapping identifizierten Angebote wurde ein erster Vorschlag zur Strukturierung der heterogenen Angebotslandschaft geschaffen, der eine vergleichende Analyse zwischen den Stadtteilen ermöglicht. Im Rahmen des Mixed-Methods-Ansatzes wurden die Ergebnisse des Online-Mappings mit den Erkenntnissen aus der qualitativen Fachkräftebefragung verglichen. Zugleich diente die Studie der methodischen Erprobung. Die Ergebnisse erlauben daher eine erste Einschätzung der Potenziale und Grenzen des gewählten methodischen Vorgehens.

Das Mapping zeigte eine deutliche sektorale Schwerpunktsetzung: In Stadtteil 1 dominierten beratende und pädagogische Angebote des Sozial- und Bildungssektors, in Stadtteil 2 hingegen teilhabeorientierte und behandlungsbezogene Angebote im Gesundheitssektor. Trotz einer höheren Einrichtungszahl in Stadtteil 2 wurden dort insgesamt weniger Angebote identifiziert – dies ist zum einen als Ressource in Stadtteil 1 interpretierbar, weist jedoch gleichzeitig auf unterschiedliche digitale Sichtbarkeit und Informationsdichte hin. In Stadtteil 2 fehlen teils Webseiten oder Detailinformationen, wodurch bestehende Angebote im Online-Desk-Research untererfasst bleiben können. Stadtteilbezogene Unterschiede können daher sowohl auf tatsächlich unterschiedliche Angebotsstrukturen als auch auf unterschiedliche digitale Webpräsentation zurückgehen.

Die Befunde deuten auf substanzielle Informations- und Zugangsbarrieren hin [12, 18]. Die Komplexität und Vielzahl der vorhandenen Angebote sowie ihre potenzielle zeitliche Dynamik erschweren sowohl Fachkräften als auch Familien den Überblick. Dies steht im Einklang zu fragmentierten Versorgungsstrukturen, unklaren Zuständigkeiten und hohen Anforderungen an die Navigationskompetenz von Familien mit chronisch kranken Kindern [19].

Zur Erhöhung der navigationalen Gesundheitskompetenz scheint es zentral, die digitale Auffindbarkeit und Aktualität von Angebotsinformationen systematisch und diversitätsorientiert zu verbessern, z. B. über standardisierte Beschreibungsformate für die Webpräsentation, um Angebote auffindbar und verstehbar zu machen sowie gleichzeitig eine bessere Patientensteuerung und Koordination zu erlauben [20–22].

Die von den Fachkräften benannten Versorgungsengstellen kontextualisieren die gefundene Angebotsstruktur. In beiden Stadtteilen werden Engpässe in der psychotherapeutischen, kinder- und jugendpsychiatrischen und -ärztlichen Versorgung sowie lange Wege zu Angeboten im Gesundheitssystem betont; im Mapping spiegelt sich dies im weitgehenden Fehlen dieser Angebote wider. Dies ist angesichts erhöhter psychosozialer Risiken in sozial benachteiligten Stadtteilen besonders problematisch [23]. Zugleich heben die Fachkräfte die Bedeutung niedrigschwelliger Freizeit‑, Sport- und Teilhabeangebote hervor, die zwar vorhanden, aber ungleich über Sektoren und Stadtteile verteilt sind.

Mit Blick auf Migration ergibt sich ein Spannungsfeld zwischen der von den Fachkräften hervorgehobenen Relevanz und einer begrenzten strukturellen Sichtbarkeit entsprechender Angebote. Fachkräfte berichten, dass Familien mit Migrationserfahrung durch die Angebote seltener erreicht werden, und sehen einen Bedarf an migrationssensibler und verständlicher Informationsvermittlung. Im Mapping sind jedoch nur wenige entsprechende Angebote erkennbar. Auch in der Funktion Teilhabe/Freizeit/offene Angebote wird Migration meist nicht explizit berücksichtigt, obwohl Kinder und Jugendliche mit Migrationserfahrung spezifische Belastungen und Ressourcen aufweisen. Zudem ist denkbar, dass lokale sozialräumliche Gegebenheiten (z. B. thematische Schwerpunkte) und institutionelle Kulturen (z. B. verwendete Fachbegriffe) beeinflussen, welche Angebote sichtbar, genutzt oder als zuständig wahrgenommen werden. Dies konnte jedoch noch nicht systematisch untersucht werden.

Insgesamt verdeutlichen Fachkräfteinterviews und Mapping, dass beide Stadtteile über eine komplexe, teils begrenzt online differenziert dargestellte Angebotslandschaft verfügen, deren Angebotsvielzahl die Orientierung zusätzlich erschwert. Für die psychosoziale Prävention erscheinen 3 Ansatzpunkte zentral:

1. Ausbau wohnortnaher kinderärztlicher, psychotherapeutischer und kinder- und jugendpsychiatrischer Angebote,
2. Erstellung von Angebotsübersichten,
3. Ausbau migrationssensibler, mehrsprachiger und teilhabeorientierter Angebote in allen Sektoren.

### Bedeutung und Nutzbarkeit des Mappings psychosozialer Angebotslandschaften

Das Sichtbarmachen der Angebotslandschaft kann eine Grundlage für den Austausch über Zuständigkeiten, Expertise und Zugangswege sowie für die sektorenübergreifende Zusammenarbeit sein. Bereits ein Überblick über alle online verfügbaren Angebote könnte Fachkräften die Weiterleitung von Klient:innen erleichtern und die sektorübergreifende Integration von Angeboten fördern, etwa im Rahmen der Schulsozialarbeit oder des Social Prescribing, also der gezielten Vermittlung in nicht medizinische Unterstützungsangebote auf Grundlage individueller sozialer und psychosozialer Bedarfe [12, 24]. Praxisnah entwickelte Präventionsketten verfolgen ähnliche Ziele, binden jedoch Gesundheitsakteure bisher nur begrenzt ein [6, 25]. Ein Mapping könnte als Informations- und Strukturierungsinstrument verstanden werden, das Transparenz erhöht und Weiterverweis und Kooperation erleichtern kann.

Gleichzeitig hängt die Steuerungsfähigkeit der Versorgung von institutionellen Rahmenbedingungen, lokalen Kulturen, (politischen) Schwerpunktsetzungen und Vergütungsstrukturen ab. Lokale Träger konkurrieren teils um Fördermittel und sind an Vorgaben der Förderlogik gebunden, etwa hinsichtlich Reichweite oder Zielgruppenerreichung [26, 27]. Dies kann eine bedarfsorientierte Weiterleitung an andere Angebote erschweren. Im Gesundheitssystem bestehen zudem nur begrenzte Anreize für sektorübergreifende Kooperation, da Koordinationsleistungen mit Akteuren außerhalb des SGB V nicht vergütet werden [28]. Eine konsequent bedarfsorientierte Steuerung wird dadurch strukturell erschwert.

Klientenseitig sind Gesundheits- und Medienkompetenz wichtige Einflussfaktoren einer verbesserten Steuerung, da sie die Fähigkeit zur Auswahl und Nutzung von Angeboten mitbestimmen [29–31]. Die kommunale Strukturentwicklung (z. B. Gesundheitskonferenzen, Angebotsübersichten wie der Präventionsnavigator Düsseldorf oder Präventionsketten) kann eine gelingende präventive Klientensteuerung fördern, indem Transparenz und Kooperationsstrukturen gestärkt werden [32–34].

Eine übersichtliche Aufbereitung vorhandener Angebote kann auch Familien dabei unterstützen, passende Hilfen zu identifizieren, und damit ihre Handlungsfähigkeit stärken [35]. Eine erhöhte Angebotstransparenz kann zudem zur navigationalen Gesundheitskompetenz beitragen, indem sie Fachkräften und Institutionen einen besseren Überblick über die lokale Angebotslandschaft ermöglicht [36]. Ob eine höhere Transparenz tatsächlich zu einer verstärkten oder bedarfsgerechteren Nutzung von Angeboten führt, lässt sich anhand der vorliegenden Daten jedoch nicht beurteilen.

### Mapping als potenziell erster Schritt hin zu besserer Patienten- bzw. Klientensteuerung

Ein Mapping könnte der erste Schritt hin zu einer verbesserten Patienten- bzw. Klientensteuerung durch „präventive Klientenpfade“ nach dem Konzept von Patient Journeys sein [19]. Solche Pfade ließen sich pro Fallkonstellation als mögliche Abfolge von Kontakten innerhalb der Angebotslandschaft konzipieren, sind bislang jedoch weder hinreichend erforscht noch sektorenübergreifend abgestimmt. Für eine effizientere Nutzung der Angebotslandschaft sind neben dem Mapping weitere Schritte notwendig, u. a. die Klärung von Zuständigkeiten mit Fachkräften vor Ort und der Abgleich der Angebotsstruktur mit valide erfassten psychosozialen Bedarfen. Wissenschaftlich wäre es zudem notwendig, Klientenpfade empirisch zu erheben (mindestens retrospektiv, besser prospektiv), um die bestehende Steuerungsqualität nachzuweisen bzw. mit fachlich konsentierten Zielpfaden zu vergleichen. Erst in Verbindung mit solchen Analysen ließe sich prüfen, inwiefern ein Angebotsmapping zu verbesserten Steuerungsprozessen, kürzeren Suchwegen oder einer passgenaueren Inanspruchnahme beiträgt.

### Stärken und Limitationen

Diese Studie ist – soweit ersichtlich – der erste systematische Versuch, die präventive psychosoziale Angebotslandschaft für Kinder im Kita- und Grundschulalter und Eltern sektorenübergreifend auf Stadtteilebene zu erfassen.

Eine Stärke liegt in der methodischen Triangulation von Fachkräfteinterviews und Online-Desk-Research. Dadurch konnten wahrgenommene Bedarfe und Zugangsbarrieren mit online sichtbaren Angebotsstrukturen in Beziehung gesetzt werden. Untersucht wurden 2 sozialstrukturell vergleichbare Stadtteile in unterschiedlichen Bundesländern mit einem hohen Anteil sozial benachteiligter Familien, wodurch sowohl fachliche Perspektiven als auch die Suchrealität von Familien und Fachkräften praxisnah abgebildet werden.

Die sektorenübergreifende Kategorisierung der identifizierten Angebote ermöglichte es, zentrale Unterstützungsfunktionen (z. B. Beratung, Alltagsunterstützung) vergleichend darzustellen. Die Kategorisierung ist jedoch nicht als validiertes Instrument zu verstehen, sondern dient der deskriptiven Systematisierung heterogener Angebotslandschaften. Zukünftige Studien sollten ihre intersubjektive Nachvollziehbarkeit, Anpassungsbedarfe und potenzielle Nutzbarkeit für Steuerungs- und Planungsprozesse weiter prüfen.

Die Angebotsidentifikation basiert überwiegend auf Internetrecherchen, sodass Angebote mit geringer Online-Präsenz vermutlich unterrepräsentiert sind. Der Abgleich mit Fachkräftebefragungen reduziert diese Verzerrung, kann sie jedoch nicht vollständig ausschließen. Zudem können Unterschiede im Detailgrad der Online-Darstellung dazu führen, dass Funktionen einzelner Angebote nur unzureichend erfasst werden. Einrichtungen außerhalb der untersuchten Stadtteile und bundesweite Online-Angebote wurden zudem noch nicht berücksichtigt.

Für die Interpretation der Ergebnisse ist zudem zu beachten, dass institutionelle Logiken und Anreizstrukturen die Sichtbarkeit und Darstellung von Angeboten beeinflussen können.

Nicht erfasst wurden bislang zentrale Aspekte wie Ressourcen und Kapazitäten (z. B. Personal, Auslastung), kulturelle Zugänglichkeit und alltagspraktische Nutzbarkeit (z. B. Erreichbarkeit, Öffnungszeiten). Die Anzahl identifizierter Angebote erlaubt daher nur eingeschränkte Aussagen zur tatsächlichen Versorgungslage; das Vorgehen bleibt explorativ.

Zudem waren die Angebote häufig altersübergreifend konzipiert, sodass die ursprünglich fokussierten Kinder im Kita- und Grundschulalter nicht immer klar abgrenzbar sind. Das Mapping bildet damit eher familienbezogene Unterstützungsstrukturen insgesamt ab. Eine Erweiterung auf Jugendliche erscheint sinnvoll, um Versorgungszuschnitte und Übergänge realitätsnäher abzubilden.

## Ausblick

Das Projekt wird in einer Ausbauphase (2025–2030) des DZPG weitergeführt und weitere Studienergebnisse liefern. Ziel ist, das Mapping in weiteren kommunalen Kontexten – insbesondere im ländlichen Raum – zu erproben und zu erweitern, die Zielgruppe auf Jugendliche auszuweiten und die Integration in bestehende Planungs- und Monitoringstrukturen zu prüfen. Die Limitationen des methodischen Vorgehens sollen im Folgeprojekt offensiv bearbeitet werden.

Dabei sollen lokale Fachkräfte noch stärker eingebunden werden – nicht nur im Rahmen von initialen Fachkräfteinterviews, sondern auch bei der Validierung und Weiterentwicklung von Angebotskategorien und der Nutzbarmachung eines Mappinginstruments über interaktive Karten, Suchportale oder Dashboards. Ein weiterentwickeltes Mapping könnte damit nicht nur der Orientierung, sondern auch als empirische Grundlage für kommunale Koordinationsprozesse dienen. Normativ stellt sich die Frage, ob das Recht von Kindern und Eltern auf Information, Zugang und Teilhabe an Unterstützungsangeboten Teil kommunaler Daseinsvorsorge sein soll und welche Rolle Mapping in dieser Debatte spielen kann.

## Supplementary Information

Onlinematerial: Funktionskategorien, sozialstrukturelle Merkmale der Stadtteile, Zitate aus Fachkräfteinterviews

## Data Availability

Die in dieser Studie generierten und analysierten Datensätze sowie die Transkripte sind wegen der Anonymität der Untersuchungsregionen nicht öffentlich verfügbar. Anfragen auf Einsicht oder Bereitstellung in geeigneter Form können an die Korrespondenzautorin gerichtet werden.

## Footnotes

1 Eine ergänzende Online-Umfrage unter Einrichtungen der Untersuchungsregionen war weniger ergiebig als das Online-Desk-Research, da vertiefende Fragen nur zu ausgewählten Angeboten gestellt werden konnten.

2 Die Anonymisierung dient dem Schutz vor einer möglichen Reidentifikation der befragten Fachkräfte.
