# Supplementary material for: Mapping municipal psychosocial services for children and parents: access and coordination barriers in preventive care—a mixed methods study
Source: Bundesgesundheitsblatt Gesundheitsforschung Gesundheitsschutz. 2026 Sep 11;69(10):1204–12. [Article in German] doi: 10.1007/s00103-026-04307-6 (PMC13624005; doi:10.1007/s00103-026-04307-6)
Supplement: Supplementary file 1 [file 103_2026_4307_MOESM1_ESM.pdf]

Onlinematerial: Funktionskategorien, sozialstrukturelle Merkmale der Stadtteile, Zitate aus Fachkräfteinterviews

Onlinematerial 1: Im Projekt entwickelte Kategorien zur Darstellung von systembezogenen Funktionen psychosozialer Angebote

1. Früherkennung und Diagnostik [Früherkennung/Diagnostik]
   Maßnahmen zur frühzeitigen Identifikation von Entwicklungsrisiken und gesundheitlichen Auffälligkeiten (z. B. Screenings, Entwicklungsdiagnostik, Vorsorgeuntersuchungen).
2. Behandlung, Gesundheits- und Entwicklungsförderung [Behandlung/Entwicklungsförderung]
   Therapeutische und förderorientierte Angebote bei Entwicklungsbedarf oder Erkrankung (z. B. Ergo-, Logo-, Psychotherapie, spezifische Förderprogramme, medizinische Nachsorge).
3. Bildung und Erziehung (vorstrukturierte Angebote) [Bildung/Erziehung]
   Vorstrukturierte Bildungs- und Lernangebote für Kinder und/oder Eltern mit klar definiertem Curriculum, festen Zeiten und Kursleitung (z. B. Nachhilfe, Elternkurse, thematische Workshops).
4. Psychosoziale Beratung [Beratung]
   Beratungsangebote zu Erziehungs-, Familien-, Gesundheits-, Bildungs- oder Rechtsfragen, einschließlich Krisen-, Sucht-, Migrations- und Teilhabeberatung.
5. Akute Intervention [Intervention]
   Angebote zur unmittelbaren Unterstützung in Krisen- und Notlagen (z. B. Krisenintervention, Notfallhilfe, Kinderschutz, Trauerbegleitung).
6. Begleitung und Alltagsunterstützung [Begleitung/Alltag]
   Hilfen zur Bewältigung des Alltags und zum Abbau von Barrieren (z. B. sozialpädagogische Familienhilfe, Begleitung zu Behörden, Unterstützung im Schulalltag, Hilfe in besonderen Lebenslagen).
7. Wohnen und Betreuung [Wohnen/Betreuung]
   Betreuungs- und Wohnformen für Kinder und Familien (z. B. Kita-Betreuung, Nachmittagsbetreuung, ambulante oder stationäre Wohn- und Betreuungsangebote).
8. Information und Aufklärung [Information/Aufklärung]
   Informations- und Sensibilisierungsangebote zu Gesundheits-, Bildungs- und Rechtsfragen (z. B. Infoabende, Broschüren, Kampagnen).
9. Vermittlung [Vermittlung]
   Angebote, die primär der Weiterleitung an andere Einrichtungen, Dienste oder Angebote dienen (z. B. Lotsenstellen, Clearingstellen, Koordinationsangebote).
10. Teilhabe, Freizeit und offene Angebote [Teilhabe/Freizeit/offene Angebote]
    Offene, niedrigschwellige Angebote zur sozialen Teilhabe, Freizeitgestaltung und Gemeinschaftsbildung (z. B. offene Treffs, Sport- und Kulturangebote, Begegnungsräume).
11. Migrations- und integrationsbezogene Unterstützung [migrationsbezogene Integration]
    Spezialisierte Angebote für Menschen mit Migrationsgeschichte, z. B. Sprach- und Integrationskurse, kultursensible Beratung, Unterstützung bei Aufenthalts- und Rechtsfragen.

12. Selbsthilfe [Selbsthilfe]
    Selbstorganisierte Gruppen und Initiativen von Betroffenen und Angehörigen (z. B. Selbsthilfegruppen zu Erziehungs-, Gesundheits- oder Belastungsthemen).

13. Sachmittel [Sachmittel]
    Materielle Unterstützungsleistungen (z. B. Kleiderkammern, Lebensmittelhilfen, Ausstattung für Schule und Freizeit).

14. Sonstige spezialisierte Angebote [Sonstiges]
    Angebote, die nicht eindeutig einer der oben genannten Kategorien zugeordnet werden konnten, aber eine psychosoziale Unterstützungsfunktion erfüllen.

Onlinematerial 2: Sozialstrukturelle Merkmale der untersuchten Stadtteile

	Stadtteil 1 (West)	Stadtteil 2 (Ost)
Anzahl Kinder im Stadtteil	~4.000	~9.000
Anteil Kinder der Gesamtstadt im Stadtteil (in %)	8 %	24 %
Anteil Kinder an Bevölkerung (in %)	18 % (Stadt: 13 %)	19 % (Stadt: 16 %)
Durchschnittsalter der Bevölkerung (in Jahren)	43 (Stadt: 44)	44 (Stadt: 45)
Anteil Deutsche mit Einwanderungsgeschichte (in %) [1]	46 % (Stadt: 35 %)	Nicht verfügbar
Anteil Bevölkerung ohne deutsche Staatsbürgerschaft (in %)	27 % (Stadt: 17 %)	32 % (Stadt: 15 %)
Anteil SGB-II-Bedarfsgemeinschaften mit Kindern an allen Haushalten mit Kindern (in %)	33 % (Stadt: 20 %)	46 % (Stadt: 19 %)

Quelle: Statistikportale der zwei Städte, Zahlen leicht gerundet

Onlinematerial 3: Zitate aus Fachkräfteinterviews

Zitat 1 (Schulpsychologische Beratungsstelle): *„Ich glaube insgesamt, es gibt so viele verschiedene Stellen und nicht jeder hat jede andere Stelle auf dem Schirm. Also ja, ne, mehr Übersicht für alle, wer was wann macht und wieso. So. Und ich glaube, Klienten blicken da zum Teil überhaupt nicht durch."*

Zitat 2 (Familienberatungsstelle): *„Mehr Bildung, mehr Aufklärung, mehr Weiterbildungen. Ich glaube, das gibt es alles schon. Da kenne ich mich einfach zu wenig in diesem System aus, um eine gute, fundierte Aussage zu treffen. Aber ich glaube, wie überall: Bildung, Aufklärung, Kennenlernen von unterschiedlichen Menschen und Kulturen würde helfen."*

Zitat 3 (Jugendhilfeeinrichtung): *„Die Familien, die wir betreuen, brauchen Unterstützung, um Hilfen zu organisieren. Das schaffen sie nicht allein, zum Beispiel ihre Kinder zu Angeboten zu bringen. Sie schaffen es nicht immer gut, sich zu informieren – obwohl das ja eigentlich heutzutage über das Internet nicht so schwierig sein dürfte."*

[1] Umfasst alle Nichtdeutschen (Ausländer*innen), Eingebürgerte, im Ausland geborene Deutsche, Aussiedler*innen sowie Minderjährige, von denen mindestens ein Elternteil eine Einwanderungsgeschichte hat (familiärer Migrationshintergrund)

Zitat 4 (Psychotherapeutin): *„Wir sind einfach zu wenige Psychotherapeut:innen, das muss man ganz klar sagen. Und vor allem in Stadtteil 2. Das ist etwas, das ich ein Stück weit als persönliche Kritik an die Kolleg:innen sehe – sie lassen sich eben gerne dort nieder, wo es schön ist, am Markt oder in Stadtteil X …“*

Zitat 5 (Jugendhilfeeinrichtung): *„Die Kinder sollten gefördert werden – und Förderung bedeutet ja nicht nur Logo- oder Ergotherapie oder ärztliche Versorgung. Für mich ist sehr wichtig, dass sie auch in ihrer allgemeinen Entwicklung gefördert werden, also durch Freizeitangebote, Sportangebote jeglicher Art. Solche Angebote gibt es, aber gerade Familien mit sehr geringem Einkommen – und das ist die Mehrzahl unserer Familien – sind darauf angewiesen, günstige oder kostenfreie Angebote zu finden, zum Beispiel über Förderungen mit 10 bis 15 Euro pro Monat. Diese Angebote sind aber auf jeden Fall nicht sehr dicht gesät.“*

Zitat 6 (Psychotherapeut): *„Was wir hier in Stadtteil X noch merken, ist, dass – obwohl die Migrationsrate recht hoch ist – migrantische Patient:innen eigentlich eher selten ankommen.“*

Zitat 7 (Schulsozialarbeit): *„Migrationshintergrund ist bei uns ein großes Thema, vor allem wegen der sprachlichen Barriere, die meist bei den Eltern liegt. Die Kinder kriegen das ja ganz schnell hin mit unserer Sprache, und wir können uns dann auch mit Händen und Füßen verständigen.“*
